# Effects of Different Rootstocks on the Physicochemical Properties and Aromatic Compounds of ‘Cabernet Sauvignon’ Wine

**DOI:** 10.3390/foods15183233

**Published:** 2026-09-13

**Authors:** Zongyi Zhang, Xiaolu Li, Wenting Zhai, Chunmei Zhu, Zongmin Fan, Zhijun Zhang, Junli Sun, Baolong Zhao

**Affiliations:** 1 Department of Horticulture, College of Agriculture, Shihezi University, Shihezi 832003, China; zhangzongyi@xjshzu.com (Z.Z.); lixiaolubit@163.com (X.L.); sjl_agr@shzu.edu.cn (J.S.); 2 The Key Laboratory of Special Fruits and Vegetables Cultivation Physiology and Germplasm Resources Utilization, Xinjiang Production and Construction Corps, Shihezi 832003, China

**Keywords:** grafting, ‘Cabernet Sauvignon’ wine, aroma compounds, aroma activity values, quality evaluation

## Abstract

This study assessed how rootstocks affect the physicochemical traits, sensory quality, and aroma profile of ‘Cabernet Sauvignon’ (CS) wine to guide rootstock selection in arid regions. Self-grafted vines (CS/CS) were the control, and five combinations (CS/5BB, CS/140R, CS/5C, CS/SO4, CS/kangzhen3) were vinified using standard procedures. Physicochemical indices, CIELab colour, and sensory attributes were evaluated; volatiles were analysed by HS-SPME-GC-MS and interpreted using PCA, OPLS-DA, and OAVs. All wines met national dry red wine standards. CS/SO4 showed the best overall physicochemical quality (composite PCA score 4.167, ranked first) and significantly higher dry extract, total sugar, soluble solids, and resveratrol than CS/CS. CS/5BB had the highest total semi-quantitative aroma compound content (53,278.32 μg·L^−1^) and the greatest ester proportion (42.85%), exceeding the other treatments (10.98–38.61%). Ninety-four volatiles were identified; 13 compounds exhibited OAV > 1, mainly esters (ethyl butyrate, ethyl hexanoate, and ethyl octanoate) and aldehydes (hexanal and heptanal) imparting fruity, floral, and green notes. OPLS-DA identified 15 discriminant compounds (VIP > 1), including ethyl acetate, phenethyl alcohol, and isopentanol. CS/5BB and CS/5C were enriched in floral/fruity esters, whereas CS/140R and CS/CS showed stronger vegetal/chemical notes. Rootstock significantly altered physicochemical properties, colour, antioxidant capacity, and aroma; CS/SO4 may improve balance and ageing potential, while CS/5BB enhances aroma complexity and fruit expression. As the data were obtained from a single vineyard site and a single harvest year, these findings require validation in multi-year trials.

## 1. Introduction

‘Cabernet Sauvignon’ (*Vitis vinifera* L. ‘Cabernet Sauvignon’) is among the most widely cultivated red grape varieties globally and holds significant economic importance in arid regions such as Xinjiang [1]. Noted for its high tannin content, high acidity, and intense aromatic potential [2,3], Cabernet Sauvignon grapes can be used to produce high-quality red wines with deep colour, rich flavour, and robust tannins [4,5]. However, wine quality is influenced by the grape’s genetic characteristics, cultivation management practices, and winemaking techniques [6]. Rootstocks indirectly affect fruit ripening, sugar and acid accumulation, and the formation of aroma precursors by regulating the scion’s water uptake, mineral nutrient supply, and hormonal signaling [7,8,9,10]. Therefore, identifying suitable rootstock–scion combinations is a crucial viticultural strategy for enhancing wine quality and diversifying wine styles. Environmental stresses in Xinjiang—such as soil salinisation, high calcium levels, and scarce precipitation—limit the growth, development, and fruit quality of this variety. Accordingly, the use of highly stress-tolerant rootstocks is considered an effective approach to mitigate these issues [8,11]. Owing to differences in genetic background, various rootstocks exhibit significant variations in the efficiency of water, nutrient, and salt uptake and utilization, which in turn lead to differences in the accumulation of sugars, acids, phenolics, and aroma precursors in the scion fruit [12,13,14]. Previous studies have shown that rootstocks can significantly influence basic quality indicators of wine, such as ethanol content, pH, total acidity, total phenols, and tannins [15,16]. However, systematic research on the effects of rootstocks on wine aroma components remains relatively limited, and comprehensive evaluations combining multivariate statistical analysis with odor activity values are lacking.

Aroma is a key determinant of wine’s sensory quality and directly influences consumer acceptance and preference [17]. Aromatic compounds in wine primarily include alcohols, esters, acids, aldehydes and ketones, and terpenes. Their sources encompass varietal aromas inherent to grape berries (e.g., methoxypyrazines and terpenes), fermentation-derived aromas produced by yeast metabolism (e.g., higher alcohols and esters), and aging-derived aromas formed during wine maturation (e.g., lactones and volatile fatty acid esters) [18]. The influence of different rootstocks on wine aroma profiles shows marked varietal specificity and chemical diversity [19,20], and these effects are strongly modulated by vintage and environmental conditions [21]. For example, 5BB rootstock significantly increases the levels of monoterpenes and C13-norisoprenoids in Cabernet Sauvignon dry red wine, thereby enhancing floral and fruity notes [22]. Similarly, SO4 rootstock increases C13-norisoprenoids, bound carbonyl compounds, and free terpenes, thereby improving aromatic complexity [23]. In addition, 1103P and 101-14 rootstocks can enhance aldehyde- and ester-associated aromas in wine [24,25]. Moreover, consumers increasingly prefer anthocyanin-rich wines because of their recognised health-promoting properties [26], and recent studies on wines and low-alcoholic fermented beverages produced from underexploited fruits, such as karonda (*Carissa carandas* L.), have highlighted the value of valorising bioactive-rich raw materials [27,28]. Therefore, systematic investigation of how different rootstocks regulate the aroma composition of ‘Cabernet Sauvignon’ wine is important for optimising scion–rootstock combinations and improving wine flavour quality.

In recent years, gas chromatography-mass spectrometry (GC-MS) combined with headspace solid-phase microextraction (HS–SPME) has been widely applied to the qualitative and quantitative characterisation of wine aroma compounds [29]. Moreover, multivariate statistical approaches, including principal component analysis (PCA), orthogonal partial least squares discriminant analysis (OPLS-DA), and odor activity value (OAV) analysis, can effectively identify key differential aroma compounds among samples and quantify their contributions to the overall aroma profile [30,31]. However, systematic investigations of the aroma composition of ‘Cabernet Sauvignon’ wines derived from different rootstock–scion combinations in arid regions remain limited, and comprehensive studies integrating physicochemical indices, phenolic composition, colour parameters, sensory evaluation, and aroma characteristics are still lacking.

In this study, self-grafted control ‘Cabernet Sauvignon’ vines were used as the control, and five representative rootstock combinations (CS/5BB, CS/140R, CS/5C, CS/SO4, and CS/kangzhen3) were established. Under the typical arid ecological conditions of Shihezi, Xinjiang, the effects of different rootstock–scion combinations on the basic physicochemical indices, sensory quality, and aroma composition of the wines were systematically evaluated. Notably, in contrast to earlier studies that focused mainly on grape composition [32,33] or on a single analytical dimension [34], the present work provides, to the best of our knowledge, the first comprehensive evaluation integrating basic physicochemical parameters, phenolic composition, CIELAB colour, sensory analysis, and HS-SPME-GC-MS-based volatile profiling (interpreted using PCA, OPLS-DA, OAV, and PLS biplot analyses) for ‘Cabernet Sauvignon’ wines from different rootstock–scion combinations under arid ecological conditions. The findings are expected to provide a preliminary reference for the rational selection of rootstocks for ‘Cabernet Sauvignon’ wine grapes in arid regions and for wine style modulation, while also enriching the theoretical understanding of how rootstock–scion interactions influence wine aroma quality; however, because the data were obtained from a single vineyard site and a single harvest year, multi-year validation will be required before firm rootstock recommendations can be made [21].

## 2. Materials and Methods

### 2.1. Experimental Site Description

The experiment was conducted in the standard grape experimental orchard at the Experimental Station of the College of Agriculture, Shihezi University (43°26′–45°20′ N, 84°58′–86°24′ E). The site has an average elevation of 450.8 m, a frost-free period of 168–171 days, an annual mean temperature of 6.5–7.2 °C, annual precipitation of 125.0–207.7 mm, and annual sunshine duration of 2721–2818 h. The soil is a multilayer sandy loam with a pH of 7.0–8.2 and is rich in calcium. The plant materials comprised ‘Cabernet Sauvignon’ scion–rootstock combinations including CS/5BB, CS/140R, CS/5C, CS/SO4, and CS/kangzhen3, with self-grafted ‘Cabernet Sauvignon’ (CS/CS) serving as the control. The characteristics of the rootstock varieties used are summarised in Table 1. All materials were planted in 2014. The vineyard was oriented east–west and trained to a low trellis with a V-shaped canopy in an inverted L-shaped row arrangement, with spacing of 3.4 m × 0.5 m between rows and vines. For each grafting combination, three vines with uniform growth and vigor were selected as experimental units, and standard vineyard management practices were applied uniformly across all treatments. The main morphological characteristics of the grape clusters and berries at harvest are summarised in Table 2.

### 2.2. Experimental Methods

#### 2.2.1. Pre-Harvest Treatment of Grapes

Starting at the onset of véraison (color development) in ‘Cabernet Sauvignon’ berries, soluble solids were measured every three days. When the mean soluble solids content of grapes from each rootstock–scion combination exceeded 20 °Brix, the fruit was considered to have reached optimal harvest maturity for winemaking. Because the combinations reached the target maturity on different dates, harvest of the six treatments occurred on successive days; the potential influence of these harvest-date differences is addressed in Section 4.4. For each combination, approximately twenty kilograms of grapes with uniform berry size were selected and thoroughly homogenised. Winemaking was performed according to the method described by Gutiérrez-Gamboa et al. [35].

#### 2.2.2. Winemaking Process

The grapes were harvested on 11–16 October 2020. The winemaking procedure was performed as follows: harvested grapes were sorted to remove stems, leaves, and unripe, diseased, mouldy, or rotten berries. All winemaking equipment was thoroughly disinfected. Subsequently, twenty kilograms of sound berries were weighed and manually crushed. The must was transferred to a 20 L ceramic jar, and SO_2_ was immediately added at 100 mg·L^−1^ to inhibit the growth of spoilage microorganisms and prevent oxidation. The maximum storage duration of the grapes between harvest and crushing was 24 h; until then, the harvested bunches were kept at 4 °C to minimise water loss and microbial spoilage. The must was then inoculated with active dry wine yeast (Saccharomyces cerevisiae, 200 mg·L^−1^; Angel Yeast Co., Ltd., Yichang, China), which was rehydrated according to the manufacturer’s instructions. Primary fermentation was conducted at 25 °C for 7 days, during which the cap was punched down twice daily (morning and evening). After pressing (the wine was separated from the skins and seeds using cheesecloth), the wine was transferred into three 3 L ceramic fermentation jars (three replicates). Secondary fermentation was conducted at 25 °C. After fermentation had ceased, the jars were sealed and allowed to clarify naturally for 40 days. The supernatant was then racked off, bottled in 750 mL green glass bottles sealed with natural cork stoppers, and aged for 12 months in a dark cellar at 15 °C. After the 12-month bottle-aging period, wine samples were collected, and the physicochemical parameters, colour, antioxidant activity, sensory attributes, and aroma compounds were determined.

#### 2.2.3. Determination of Basic Physicochemical Parameters of Wine

Basic physicochemical parameters of wines from different scion–rootstock combinations—including alcohol content, dry extract, volatile acidity, total sugar, total acidity, and total SO_2_—were quantified according to GB/T 15038-2006 (“General Analytical Methods for Wine and Fruit Wine”) [36]. Soluble solids were measured using a PAL-1 handheld refractometer (ATAGO Co., Ltd., Tokyo, Japan), and pH was determined with a pH meter (Shanghai Leici Instrument Co., Ltd., Shanghai, China). Total phenolics were determined by the Folin–Ciocalteu method [37]; tannins by the Folin-Denis method [26]; anthocyanins by the pH-differential method [38]; and total flavonoids by the NaNO_2_-AlCl_3_ colorimetric method [39]. Resveratrol was quantified by ultra-high-performance liquid chromatography (UPLC) [32]. Reduced ascorbic acid (Vc) and total antioxidant capacity (TAC) were determined using commercial assay kits (Coolaber Science & Technology Co., Ltd., Beijing, China). CIELAB colour parameters were determined following the method of Pérez et al. [40]. All measurements were performed in triplicate.

#### 2.2.4. Sensory Evaluation of Wine

Four nationally certified wine tasters from Xinjiang CITIC Guoan Wine Industry Co., Ltd. (Nascent Branch, Manas County, Changji Hui Autonomous Prefecture, Xinjiang, China) evaluated the wines according to established criteria, scoring colour, clarity, aroma, taste, and typicity. The resulting scores were subjected to statistical analysis and comprehensive comparison.

#### 2.2.5. Determination of Aromatic Compounds in Wine

In this study, headspace solid-phase microextraction (HS-SPME) was employed to extract wine aroma compounds. Briefly, 5 mL of sample was transferred into a 20 mL headspace vial, followed by the addition of 1 g NaCl, 2 μL of 2-octanol (825 μg·L^−1^; internal standard), and a magnetic stir bar. The vial was sealed immediately, shaken, and placed on a thermostated magnetic stirrer at 45 °C to equilibrate for 10 min. A preconditioned SPME fiber (50/30 μm divinylbenzene/carboxen/polydimethylsiloxane, DVB/CAR/PDMS; Supelco, Bellefonte, PA, USA) was then exposed to the headspace for 40 min at 45 °C. After extraction, the fiber was withdrawn and immediately introduced into the GC injector, where desorption was carried out at 250 °C for 5 min as data acquisition commenced. The fiber was removed after 5 min. The GC oven program was as follows: 40 °C for 5 min; ramped at 3 °C·min^−1^ to 100 °C; ramped at 5 °C·min^−1^ to 180 °C; ramped at 20 °C·min^−1^ to 230 °C and held for 5 min. The total run time was 48.5 min.

#### 2.2.6. Chromatography-Mass Spectrometry Conditions

Chromatographic conditions were as follows: an HP-INNOWAX capillary column (60 m × 0.25 mm × 0.25 μm) was used with high-purity helium (99.999%) as the carrier gas at a flow rate of 1 mL·min^−1^, and the injector temperature was set to 250 °C.

MS conditions were as follows: the interface temperature was 280 °C, the ion source temperature was 230 °C, the quadrupole temperature was 150 °C, ionization was performed in electron impact (EI) mode at 70 eV, and mass spectra were acquired over an *m*/*z* range of 20–350.

#### 2.2.7. Data Analysis

Data processing: Three independent experiments were performed in triplicate. The resulting data were analysed by analysis of variance (ANOVA) and are reported as the mean ± standard deviation (SD). Statistical significance was determined using SPSS 26.0 and Excel 2019.

Qualitative analysis of aroma components: Compounds were tentatively identified by matching the acquired mass spectra against the Wiley and NIST 17 libraries embedded in the instrument software. Each identification was further confirmed by comparing the experimentally determined linear retention index (LRI) with literature values reported for polar (INNOWAX-type) columns. LRIs were calculated from the retention times of a homologous series of n-alkanes (C6–C30) analysed under identical chromatographic conditions, according to the equation of van Den Dool and Kratz [41]. Only compounds for which both the mass-spectral match and the LRI comparison were consistent were considered identified; compounds whose physico-chemical properties are incompatible with HS-SPME-GC-MS analysis (e.g., essentially non-volatile substances or synthetic additives such as UV filters) were excluded from the final dataset. The LRI of every identified compound is provided in Supplementary Table S1.

Semi-quantitative analysis of aroma compounds: Because HS-SPME is an equilibrium-based extraction technique in which the wine matrix and ethanol content markedly affect the distribution of analytes among the liquid phase, the headspace, and the fiber, an internal standard alone does not guarantee fully accurate quantification; the values obtained here therefore represent relative semi-quantitative contents rather than absolute concentrations [42]. Semi-quantitative contents were estimated by the internal standard method using 2-octanol as the internal standard, and all between-treatment comparisons were made on this basis. The content of each aroma compound was calculated according to the following equation:

Content of analyte = V1/V2 × C

where;

V1—peak area of the analyte;
V2—Peak area of 2-octanol;
C—concentration (μg·L^−1^) of the internal standard 2-octanol.

#### 2.2.8. Microbiological Evaluation

To evaluate the microbiological stability of the wines and any spoilage by food-borne microorganisms during storage, wine samples were analysed at the end of the 12-month bottle-aging period. Total aerobic mesophilic bacteria, yeasts and moulds, and lactic acid bacteria were enumerated by standard plate-count methods on plate count agar (30 °C, 48 h), potato dextrose agar (25 °C, 5 d), and MRS agar (30 °C, 5 d), respectively.

## 3. Results

### 3.1. Effects of Different Rootstocks on the Physicochemical Properties of ‘Cabernet Sauvignon’ Wine

#### 3.1.1. Effects of Different Rootstocks on the Basic Components of ‘Cabernet Sauvignon’ Wine

**Figure 1:**
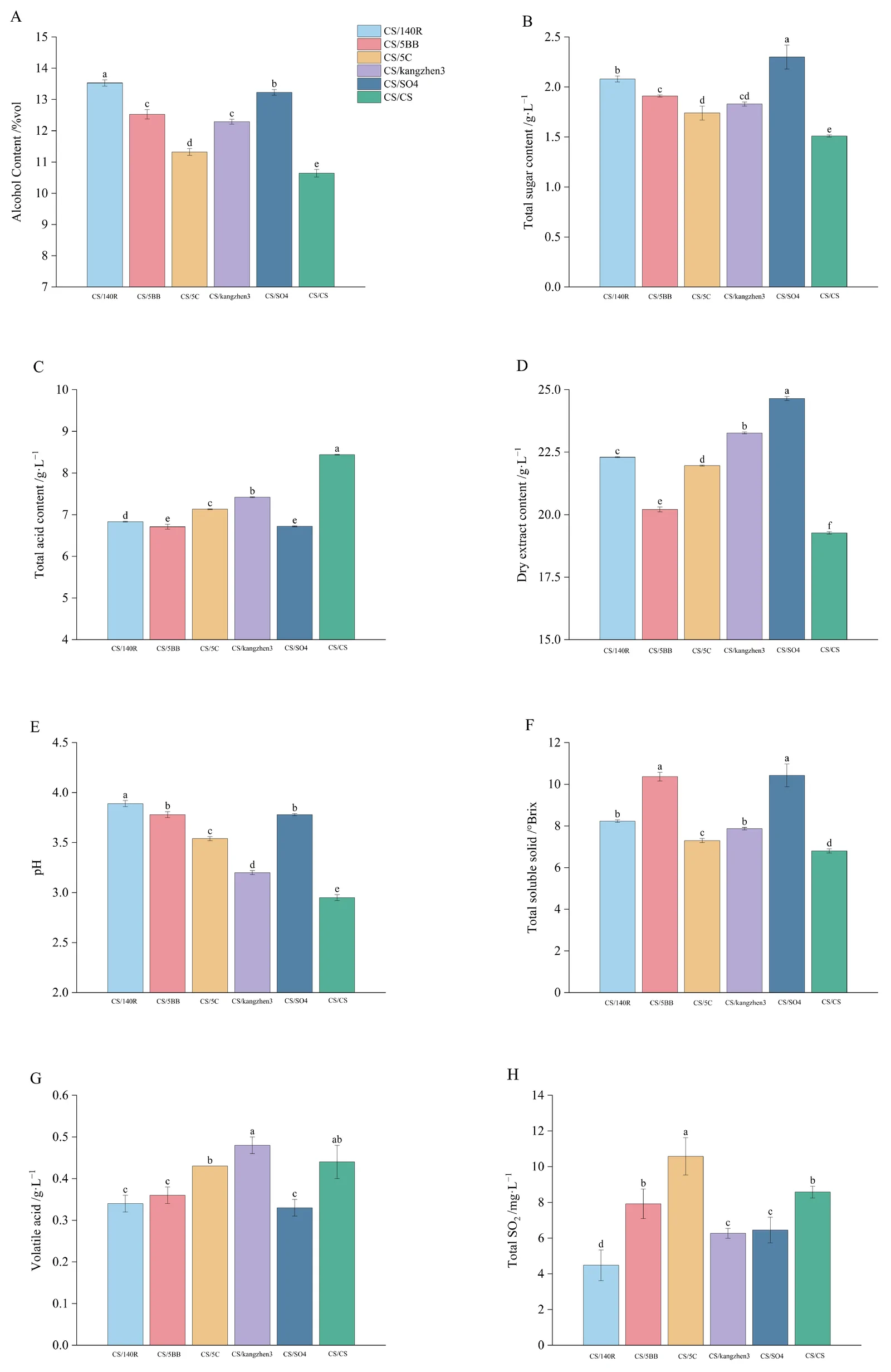
Effects of different rootstocks on conventional physicochemical indices of ‘Cabernet Sauvignon’ wine. (**A**): alcohol content; (**B**): total sugar content; (**C**): total acid content; (**D**): dry extract content; (**E**): pH; (**F**): total soluble solids; (**G**): volatile acidity; (**H**): total SO_2_. Note: Different letters indicate significant differences among treatments (Tukey’s test, *p* < 0.05). Values are means ± SD (*n* = 3). The same notation applies to the subsequent figures.

All physicochemical and bioactive parameters reported in this section were determined on wine sampled at the end of the 12-month bottle-aging period (approximately 14 months after the harvest on 11–16 October 2020). As illustrated in Figure 1, the various rootstocks exerted distinct effects on the physicochemical attributes of ‘Cabernet Sauvignon’ wine. Fermentation proceeded to near completion across all treatments: dry extract values consistently exceeded 18.0 g·L^−1^, alcohol levels were uniformly above 7.0% (*v*/*v*), and total sugar remained below 4 g·L^−1^. All parameters satisfied the physicochemical criteria for dry red wine stipulated in the National Mandatory Standard for Wine (2018 edition), thereby confirming that every sample qualified as a dry red wine. In addition, microbiological evaluation of the bottled wines at the end of storage showed that all wines complied with the specified microbiological limits: total aerobic mesophilic bacteria < 10 CFU·mL^−1^, yeasts and moulds < 10 CFU·mL^−1^, and lactic acid bacteria < 10 CFU·mL^−1^ in every treatment, confirming that no spoilage by food-borne microorganisms occurred during the 12-month storage period (Table S2).

As shown in Figure 1A, alcohol content peaked in the CS/140R combination. No significant difference was observed between CS/5BB and CS/kangzhen3, whereas significant differences were evident among the other combinations. As shown in Figure 1B, total sugar was highest in CS/SO4, significantly exceeding that of all other rootstock–scion combinations; conversely, CS/CS exhibited the lowest total sugar, significantly lower than the remaining combinations.

In wine, acid concentration is a principal determinant of perceived acidity. Insufficient acidity dulls flavour complexity and diminishes ageing capacity; excessive acidity, by contrast, readily destabilises the structural balance, producing a thin or abrasive palate. In general, at an acidity of 2 g·L^−1^ the wine tastes conspicuously flat, whereas at 10 g·L^−1^ it becomes markedly sharp. Accordingly, an optimal acidity is typically maintained within 4–8 g·L^−1^ to preserve sensory equilibrium and quality stability [43]. As shown in Figure 1C, total acidity was highest in the CS/CS combination, significantly exceeding that of all other treatments, and lowest in CS/5BB. No significant difference was detected between CS/5BB and CS/SO4, while the remaining combinations differed significantly from one another.

Dry extract is a pivotal metric for evaluating wine quality, stylistic expression, and vinification practice. Elevated dry extract generally corresponds to higher perceived quality, a more substantial mouthfeel, greater flavour complexity, and enhanced ageing potential. As shown in Figure 1D, dry extract differed significantly among treatments, with CS/SO4 exhibiting the highest level and CS/CS the lowest. As shown in Figure 1E, pH ranged from 2.98 to 3.89 across the combinations. No significant difference was observed between CS/5C and CS/SO4; however, both differed significantly from the other four rootstock–scion combinations. The CS/140R combination displayed the highest pH, whereas CS/CS had the lowest.

Soluble solids provide a direct measure of grape maturity and sugar accumulation at harvest, forming the basis for predicting both the wine’s eventual alcohol level and its stylistic potential. As shown in Figure 1F, the CS/SO4 combination exhibited the highest soluble-solids content, with no significant difference from CS/5BB. Both were significantly higher than the remaining combinations, implying a greater capacity to accumulate flavour precursors and related compounds. By contrast, CS/CS showed the lowest soluble-solids content, significantly below that of all other treatments.

Volatile acidity in ‘Cabernet Sauvignon’ wine is a critical index of quality and of the integrity of the vinification process; in general, lower volatile acidity corresponds to a cleaner, purer profile. As shown in Figure 1G, CS/140R, CS/5BB, and CS/SO4 displayed relatively low volatile acidity, significantly lower than the other three rootstock–scion combinations, indicating that these wines were comparatively purer. Sulfur dioxide (SO2), as an antioxidant, suppresses microbial growth and helps prevent spoilage. As shown in Figure 1H, although SO2 levels in all six combinations complied with national standards, CS/5C had the highest SO2 content, significantly exceeding that of the other treatments, suggesting a potentially stronger antioxidative capacity.

#### 3.1.2. Effects of Different Rootstocks on Phenolic Compounds in ‘Cabernet Sauvignon’ Wine

**Figure 2:**
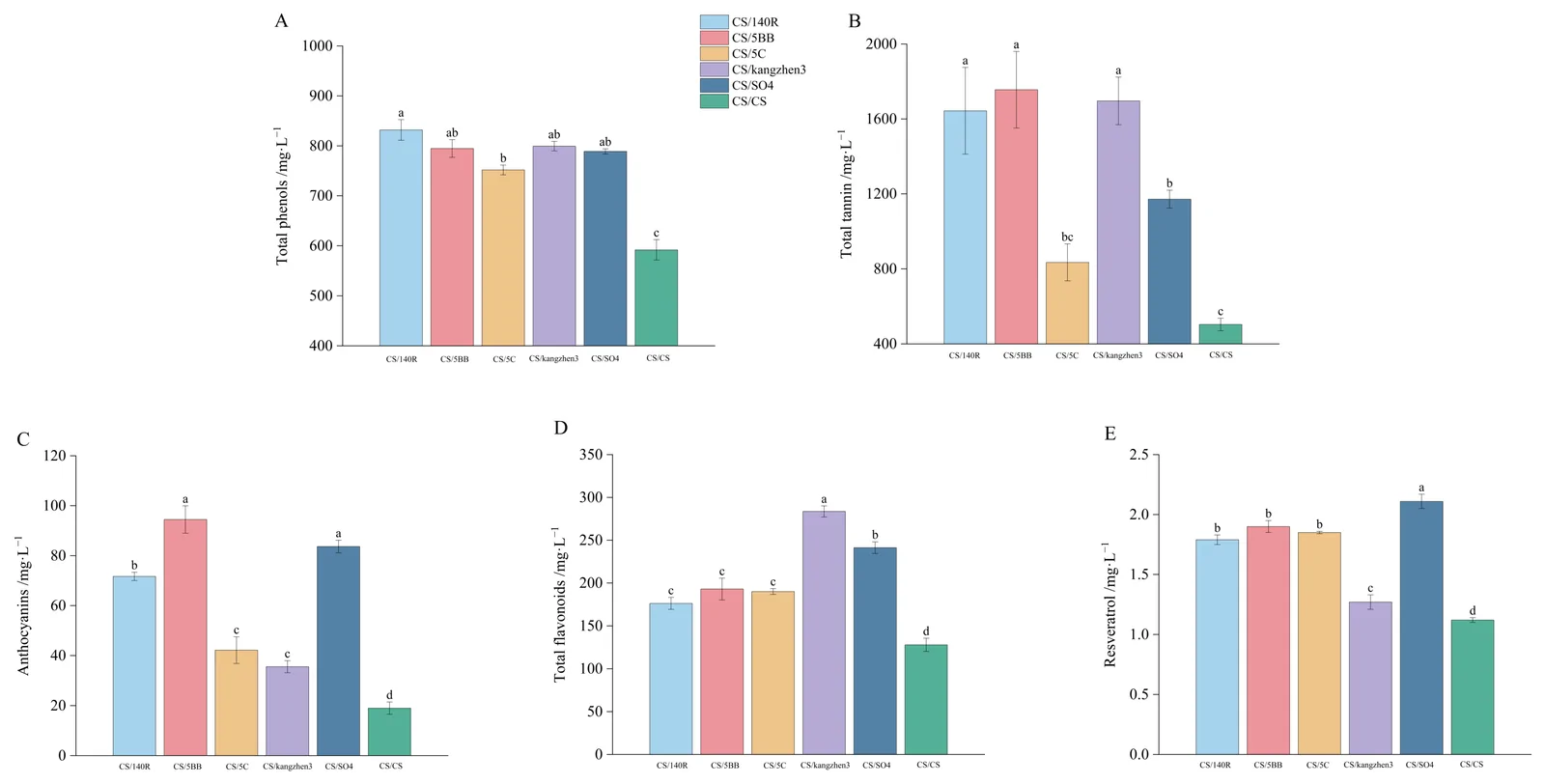
Effects of different rootstocks on phenolic compounds in ‘Cabernet Sauvignon’ wine. (**A**): total phenols; (**B**): total tannins; (**C**): total anthocyanins; (**D**): total flavonoids; (**E**): resveratrol.

Phenolic compounds are pivotal determinants of red wine colour, flavour, structure, and antioxidant capacity; collectively, they shape overall quality, ageing potential, and certain putative health benefits. As shown in Figure 2, relative to the control, all five rootstocks significantly increased the concentrations of total phenolics, tannins, total anthocyanins, total flavonoids, and resveratrol in ‘Cabernet Sauvignon’ wine. In general, elevated tannin levels are indicative of strong ageing aptitude. In the present study, the CS/140R, CS/5BB, and CS/kangzhen3 combinations exhibited higher tannin contents, significantly surpassing those of the other rootstock–scion combinations. Because anthocyanins function as antioxidants, higher anthocyanin concentrations also imply a greater colour-imparting capacity. Anthocyanin content in CS/5BB and CS/SO4 was significantly higher than in the other combinations, suggesting that these wines possess superior antioxidative and colour-imparting potential. Total flavonoids constitute a broader, more integrative indicator than either tannins or anthocyanins: they reflect antioxidant capacity while also contributing to palatal complexity. Total flavonoid content in CS/kangzhen3 and CS/SO4 was significantly higher than in the remaining combinations, implying stronger antioxidant potential and enhanced flavour quality. Resveratrol, an anti-inflammatory and antioxidant compound, is associated with potential health benefits. In this study, resveratrol content was highest in CS/SO4 and significantly greater than in the other rootstock–scion combinations, indicating a comparatively higher health-promoting potential.

#### 3.1.3. Effects of Different Rootstocks on the Total Antioxidant Capacity and Vitamin C Content of ‘Cabernet Sauvignon’ Wine

**Figure 3:**
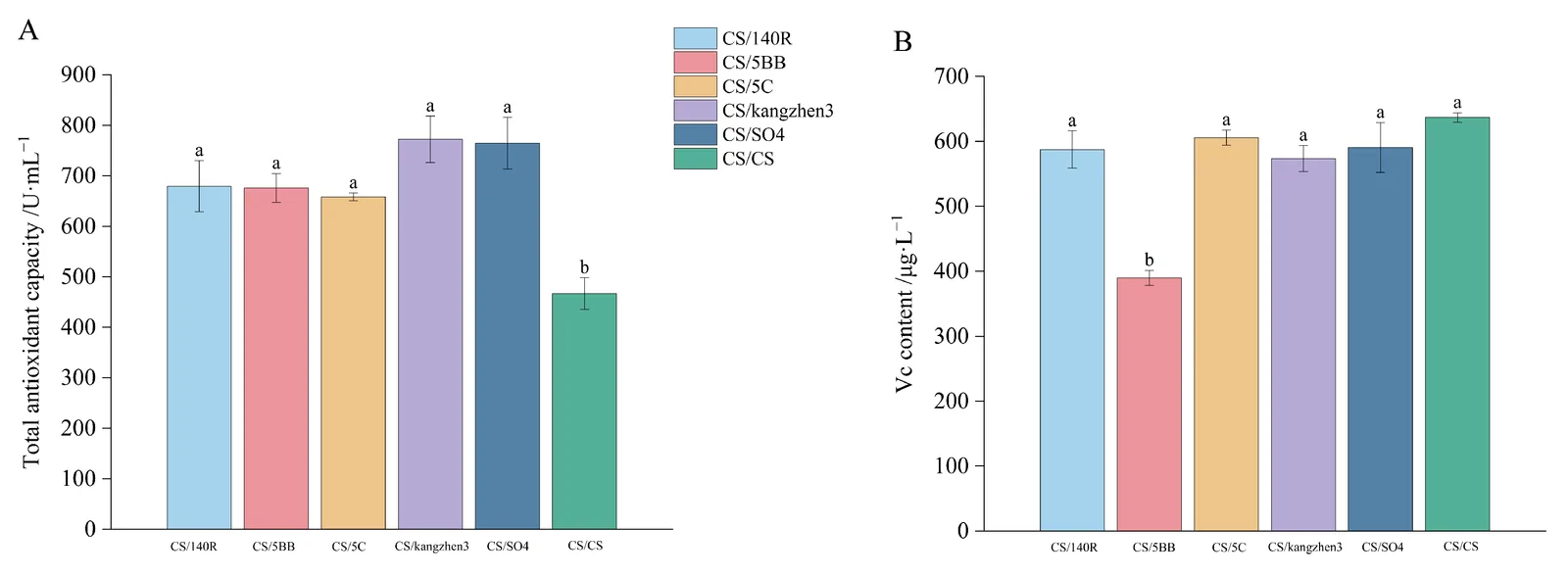
Effects of different rootstocks on the antioxidant capacity and vitamin C content of ‘Cabernet Sauvignon’ wine. (**A**): total antioxidant capacity; (**B**): vitamin C (Vc) content.

Total antioxidant capacity denotes a wine’s integrated ability to neutralise free radicals and withstand oxidative degradation. As shown in Figure 3, all five rootstocks significantly enhanced the wine’s total antioxidant capacity. Vitamin C is a potent reducing agent that preferentially reacts with dissolved oxygen in wine, thereby safeguarding fruit-derived aromas. Among ‘Cabernet Sauvignon’ wines produced on different rootstocks, the CS/5BB treatment exhibited a significantly lower vitamin C content than the other treatments, whereas no significant differences were detected among the remaining treatments.

#### 3.1.4. Comprehensive Evaluation of the Physicochemical Quality of ‘Cabernet Sauvignon’ Wine from Different Rootstocks

Principal component analysis (PCA) was conducted on 15 physicochemical attributes of ‘Cabernet Sauvignon’ wines produced on different rootstocks (Table 3). The eigenvalues of the first four principal components were 9.014, 2.308, 1.434, and 1.128, respectively. Collectively, these components explained 92.564% of the total variance, indicating that they capture most of the information contained in the dataset. In the first principal component, pH, anthocyanins, resveratrol, and volatile acidity made the largest contributions; in the second principal component, dry extract, total flavonoids, and total antioxidant capacity contributed most strongly; in the third principal component, total SO2 was the dominant contributor; and in the fourth principal component, vitamin C content contributed most substantially.

According to the comprehensive score rankings (Table 4), the overall performance of ‘Cabernet Sauvignon’ wines across rootstocks, from highest to lowest, was as follows: CS/SO4 > CS/5BB > CS/140R > CS/kangzhen3 > CS/5C > CS/CS.

### 3.2. Effects of Different Rootstocks on the Sensory Quality of ‘Cabernet Sauvignon’ Wine

#### 3.2.1. Effects of Different Rootstocks on the Color of ‘Cabernet Sauvignon’ Wine

Wine colour is a pivotal sensory and physicochemical index for assessing varietal expression, ripeness, vinification practices, and ageing status, and it is closely tied to consumer acceptance and perceived quality. Luminance (L*) denotes wine brightness. As shown in Table 5, the tested wines exhibited moderate brightness. The CS/CS combination showed the highest L* value, significantly exceeding those of the other rootstock–scion combinations, whereas CS/SO4 displayed the lowest L* value, significantly below the others.

The a* value reflects the red-green dimension of the sample: values from −100 to +100 indicate a shift from greener to redder tones. Among the treatments, CS/SO4 presented the highest a* value, significantly higher than the other combinations, while CS/CS had the lowest a* value, significantly lower than the remainder.

The b* value represents the yellow-blue dimension, also ranging from −100 to +100, with higher values indicating increased yellowness and reduced blueness. CS/SO4 exhibited the highest b* value, significantly higher than the other combinations, whereas CS/kangzhen3 recorded the lowest b* value, significantly lower than the others.

Chroma (c*) indicates colour saturation, describing the intensity or concentration of colour within a given hue. CS/SO4 showed the highest c* value among the tested wines, suggesting superior colour saturation. The hue angle (*h*°) is another key colour descriptor; for wines, *h*° typically falls between 0° and 90°. In this study, *h*° ranged from 16.29° to 20.18°, indicating hues close to brick red and tile red. CS/5BB exhibited the highest *h*° value, whereas CS/kangzhen3 showed the lowest.

The overall colour difference (Δ*E*) quantifies the magnitude of colour divergence among samples. Using CS/CS as the reference, the other samples displayed relatively small deviations. The largest Δ*E* was observed for CS/SO4 (11.91), while the smallest occurred in CS/kangzhen3 (5.46).

**Figure 4:**
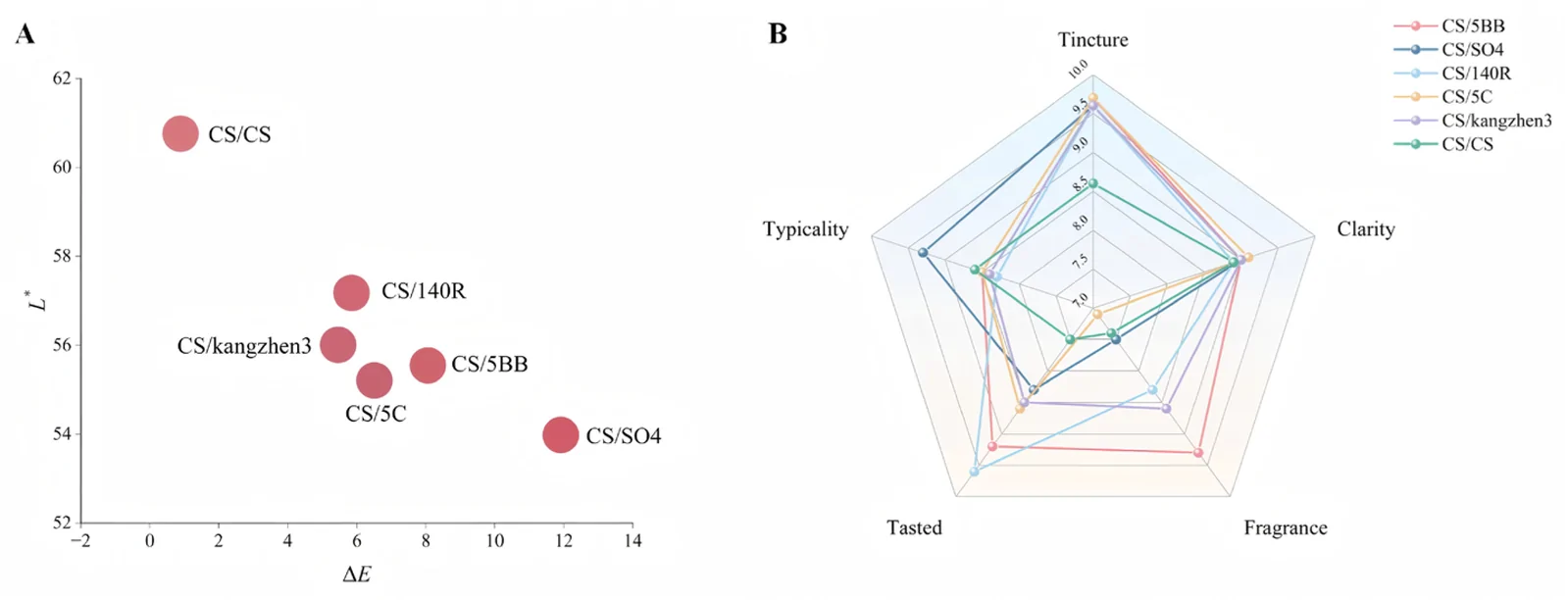
Characteristic colours and sensory evaluation of ‘Cabernet Sauvignon’ wines from different rootstocks. (**A**): characteristic colours of the wines; (**B**): radar chart of sensory evaluation.

Conventional CIELAB colour analysis merely yields parameter values and does not directly convey the characteristic hues, making it difficult to characterise wine colour in an intuitive manner. As illustrated in Figure 4A, each wine sample is denoted by a numbered circular marker. According to their spatial distribution, the Δ*E* and L* values were used as the x- and y-coordinates, respectively, and the corresponding colours were assigned to the markers, thereby visualising the characteristic colour of each wine sample. The CS/CS wine displayed high brightness but low saturation, presenting as a rose-pink hue; by contrast, the CS/SO4 wine was darker yet exhibited a more pronounced red tone and higher saturation, appearing brick red.

#### 3.2.2. Sensory Evaluation of ‘Cabernet Sauvignon’ Wines from Different Rootstocks

As shown in the radar plot (Figure 4B) and the scoring matrix (Table 6), sensory assessment of ‘Cabernet Sauvignon’ wines grafted onto different rootstocks revealed discernible differences. For colour, CS/5BB and CS/5C achieved the highest scores, whereas CS/CS scored lowest; for clarity, CS/5C ranked highest, while CS/140R and CS/CS ranked lowest; for aroma, CS/5BB ranked highest, while CS/5C ranked lowest; for mouthfeel, CS/140R ranked highest, whereas CS/CS ranked lowest; and for typicity, CS/SO4 ranked highest, while CS/140R ranked lowest.

Integrating colour, clarity, aroma, mouthfeel, and typicity, the overall sensory quality of the wines was ranked as follows: CS/5BB > CS/140R > CS/kangzhen3 > CS/SO4 > CS/5C > CS/CS. In terms of global appraisal, the CS/SO4 wine exhibited a well-proportioned palate, whereas the CS/5BB and CS/140R wines appeared poorly balanced. The CS/5C, CS/kangzhen3, and CS/CS wines were marked by pronounced volatile acidity and chlorinous notes.

### 3.3. Effects of Different Rootstocks on the Aromatic Compounds of ‘Cabernet Sauvignon’ Wine

#### 3.3.1. Effects of Different Rootstocks on Alcoholic Compounds in ‘Cabernet Sauvignon’ Wine

**Figure 5:**
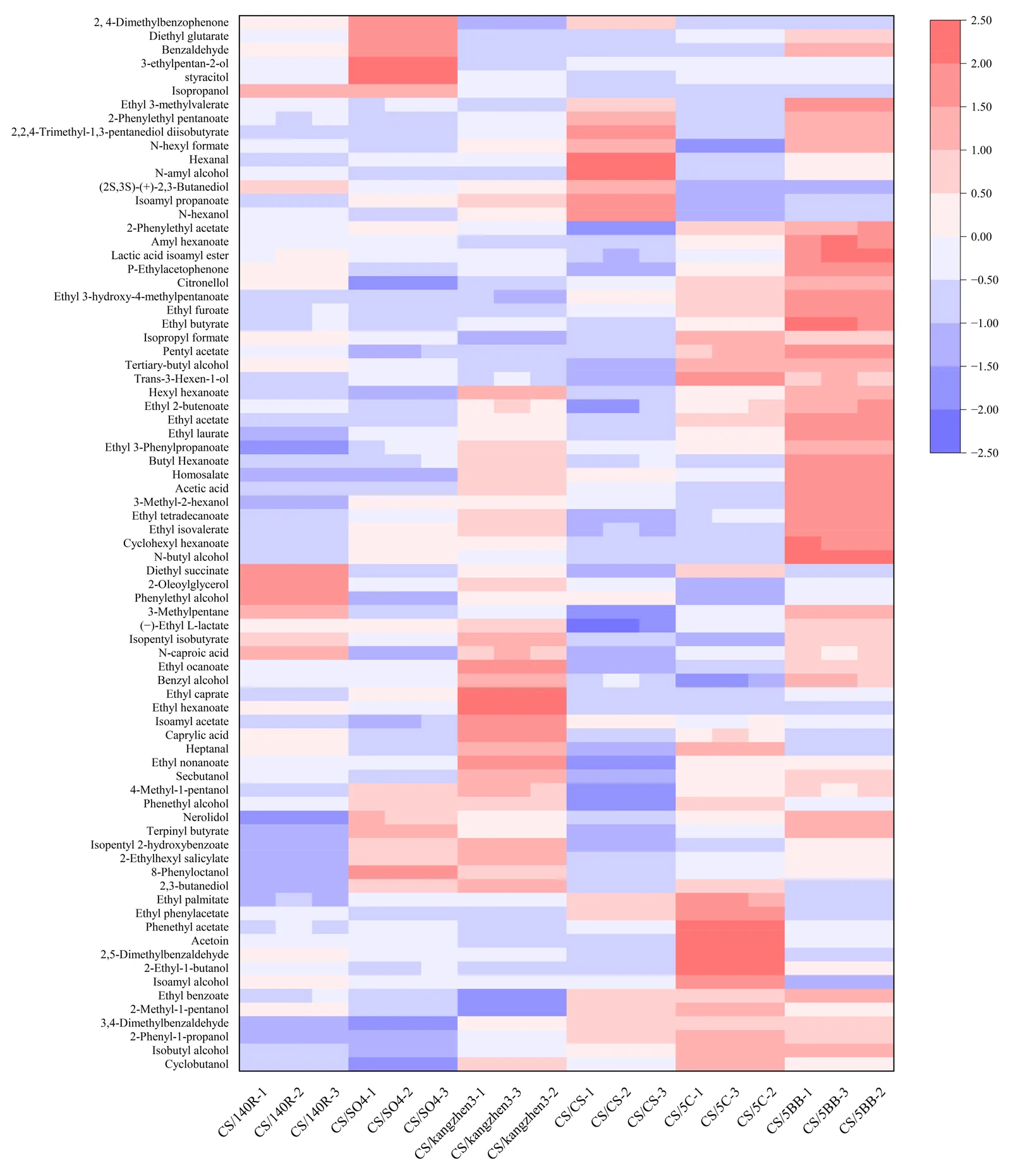
Heatmap of semi-quantitative aroma compound content in ‘Cabernet Sauvignon’ wine grafted onto different rootstocks.

As shown in Figure 5, a total of 23 alcohols were identified in this study. Across the six rootstock–scion combinations, 12 alcohols were present at semi-quantitative contents exceeding 100 μg·L^−1^, and three exceeded 1000 μg·L^−1^ (Supplementary Table S1). Notably, CS/140R exhibited the highest phenethyl alcohol content, significantly greater than that of all other combinations. CS/CS showed the highest contents of n-hexanol, n-pentanol, and (2S,3S)-(+)-2,3-butanediol. CS/5BB contained the greatest amounts of isobutanol, n-butanol, and tert-butanol, while CS/kangzhen3 likewise displayed the highest level of n-butanol. CS/SO4 recorded the highest contents of isopropanol and 3-ethyl-2-pentanol. CS/5C had the highest levels of cyclobutanol, isopentanol, 2-phenylethanol, and 2-methyl-1-pentanol.

#### 3.3.2. Effects of Different Rootstocks on Aldehyde and Ketone Compounds in ‘Cabernet Sauvignon’ Wine

As shown in Figure 5, five aldehydes and three ketones were detected in this experiment (Supplementary Table S1). Among the aldehydes, n-hexanal was most abundant in the CS/CS combination and least abundant in CS/5C. Heptanal reached its highest level in CS/5C, significantly exceeding that in the other combinations. With respect to ketones, CS/5C exhibited the highest hydroxybutanone content, differing significantly from the other combinations. The CS/5BB combination contained the greatest amount of p-ethylacetophenone, whereas CS/CS contained the least.

#### 3.3.3. Effects of Different Rootstocks on Acidic Compounds in ‘Cabernet Sauvignon’ Wine

As shown in Figure 5, three acids—acetic acid, n-hexanoic acid, and octanoic acid—were detected in ‘Cabernet Sauvignon’ wines grafted onto different rootstocks. All three occurred at contents exceeding 10 μg·L^−1^ (Supplementary Table S1). Acetic acid was significantly higher in the CS/5BB combination than in the others. n-Hexanoic acid was highest in CS/140R, significantly surpassing the remaining rootstock–scion combinations. Octanoic acid reached its greatest concentration in CS/kangzhen3, again significantly higher than in the other combinations.

#### 3.3.4. Effects of Different Rootstocks on Ester Compounds in ‘Cabernet Sauvignon’ Wine

As shown in Figure 5, a total of 41 ester compounds were identified in wines produced from different ‘Cabernet Sauvignon’ scion–rootstock combinations (Supplementary Table S1). Most of these esters are chiefly associated with floral and fruity aromatic notes. Four esters exceeded 500 μg·L^−1^: diethyl succinate, isopropyl formate, ethyl acetate, and L-lactyl acetate. Thirty-one compounds ranged from 10 to 500 μg·L^−1^, whereas all remaining components were below 10 μg·L^−1^.

Ethyl acetate was most abundant in CS/5BB, significantly surpassing the other rootstock–scion combinations. Diethyl succinate contents, in descending order, were CS/140R > CS/5C > CS/kangzhen3 > CS/SO4 > CS/5BB > CS/CS, with significant differences among combinations. Isopropyl formate exceeded 500 μg·L^−1^ in every combination; CS/5C exhibited the highest level, significantly greater than those of the other combinations. L-lactyl acetate was highest in CS/5BB and lowest in CS/CS.

#### 3.3.5. Effects of Different Rootstocks on Terpene Aroma Compounds in ‘Cabernet Sauvignon’ Wine

As shown in Figure 5, two terpenoid compounds were detected in the wine samples, and both occurred at contents exceeding 50 μg·L^−1^ (Supplementary Table S1). Citronellol and nerolidol were most abundant in the CS/5BB combination, significantly higher than in the other combinations. Citronellol levels differed significantly among the six rootstock–scion combinations, whereas nerolidol showed no significant difference between CS/5BB and CS/kangzhen3.

#### 3.3.6. Effect of Different Rootstocks on the Total Semi-Quantitative Aroma Compound Content of ‘Cabernet Sauvignon’ Wine

**Figure 6:**
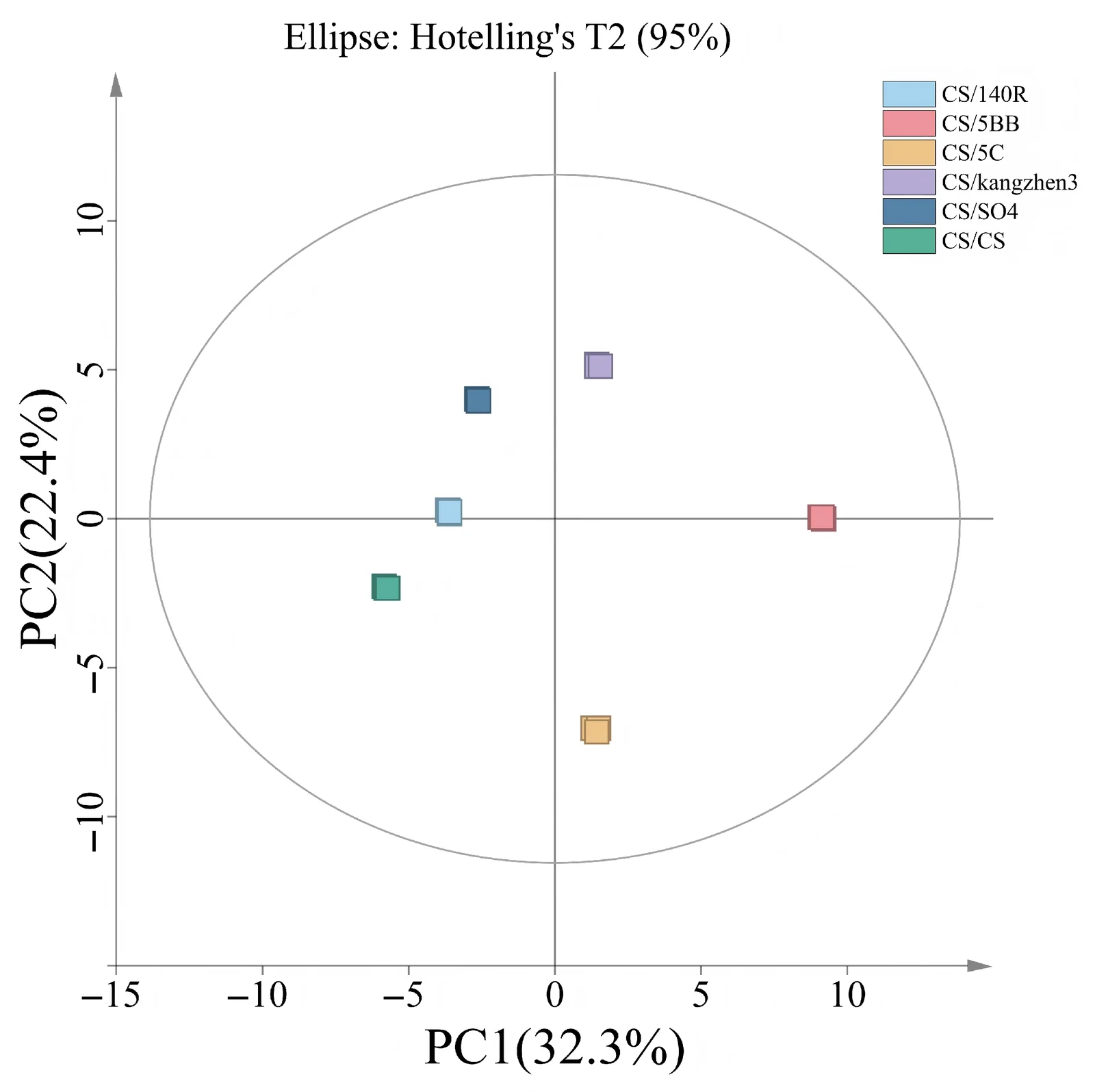
Principal component analysis of aroma components in ‘Cabernet Sauvignon’ wine grafted onto different rootstocks.

Figure 6 presents a principal component analysis (PCA) of aroma constituents in ‘Cabernet Sauvignon’ wines produced from different rootstocks. Principal Component 1 (PC1) and Principal Component 2 (PC2) together explain 54.7% of the total variance, and all combinations lie within the 95% confidence ellipse. The CS/5BB combination is clearly separated along PC1, whereas CS/5C shows marked separation along PC2.

As shown in Table 7, six classes of aroma compounds were detected across the various rootstock–scion combinations. CS/5BB exhibited the highest total semi-quantitative aroma compound content. Total semi-quantitative contents in the remaining combinations increased in the following order: CS/SO4, CS/CS, CS/5C, CS/kangzhen3, and CS/140R. Alcohols constituted the dominant fraction in all treatments (50.32–84.09%), underscoring their central contribution to overall aroma. The CS/CS treatment had the highest proportion of alcohols (84.09%), while CS/5BB had the lowest (50.32%). Notably, CS/5BB displayed the greatest ester proportion (42.85%) among the six treatments, indicating that CS/5BB substantially elevated the ester share while simultaneously enhancing total semi-quantitative aroma compound content.

#### 3.3.7. OPLS-DA Analysis of Aromatic Compounds in ‘Cabernet Sauvignon’ Wines from Different Rootstocks

**Figure 7:**
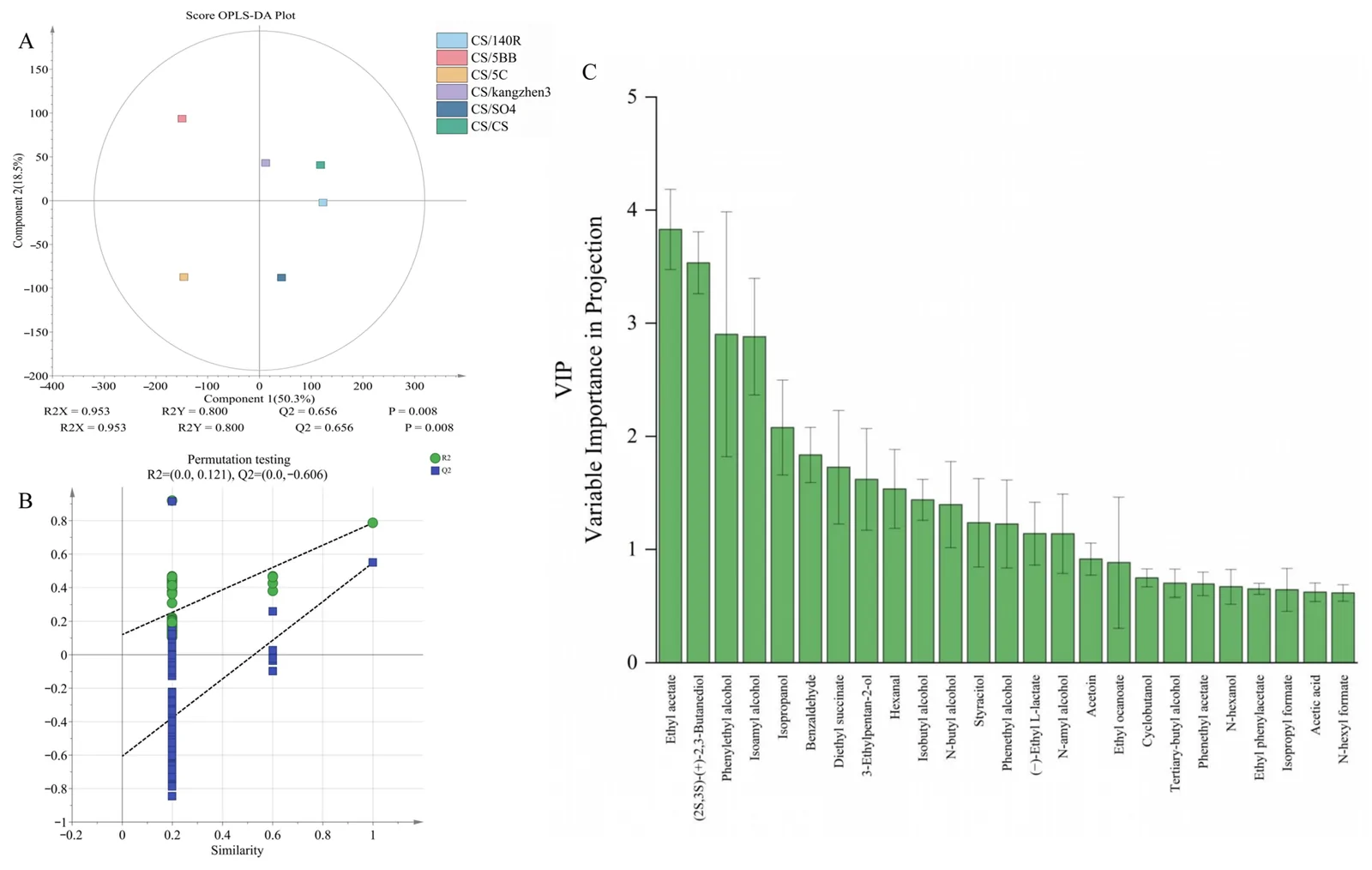
OPLS-DA analysis and VIP scores of aroma components in ‘Cabernet Sauvignon’ wine grafted onto different rootstocks. (**A**): OPLS-DA score plot; (**B**): permutation testing; (**C**): VIP values (top 25). Note: The dashed lines represent the regression trend lines of the permutation test.

With 94 aroma compounds treated as dependent variables and ‘Cabernet Sauvignon’ wines from different rootstock–scion combinations as the grouping factor, OPLS-DA clearly discriminated among the six samples (Figure 7A). All samples were encompassed within the 95% confidence ellipse. The model exhibited strong performance, with R2X = 0.953, R2Y = 0.800, and Q2 = 0.656. As both R2 and Q2 exceeded 0.5, the model displayed a robust, statistically meaningful fit and satisfactory predictive capacity.

To evaluate potential overfitting, a 200-iteration permutation test was conducted. Both model components were subjected to 200 permutations (Figure 7B). The intercepts for R2 and Q2 were 0.121 and −0.606, respectively, indicating no overfitting. Collectively, these findings support the use of this model for discriminant analysis of wine aroma profiles.

The variable importance in projection (VIP) score is a weighted index within the OPLS-DA framework that quantifies each volatile compound’s contribution to sample discrimination. Figure 7C presents the top 25 VIP-ranked volatiles in ‘Cabernet Sauvignon’ wines grafted onto different rootstocks; higher VIP scores denote more pronounced differences among the wine samples.

As shown in Figure 7C, the following compounds all exhibited VIP values > 1: ethyl acetate, (2S,3S)-(+)-2,3-butanediol, phenethyl alcohol, isopentanol, isopropanol, benzaldehyde, diethyl succinate, 3-ethyl-2-pentanol, n-hexanal, isobutanol, n-butanol, sulcatol, 2-phenylethanol, L-lactyl acetate, and n-amyl alcohol. These volatiles were strongly associated with wines from different rootstocks and can therefore be regarded as characteristic marker compounds for classification.

Among them, ethyl acetate displayed the highest VIP value (3.83), followed by (2S,3S)-(+)-2,3-butanediol (3.53), phenethyl alcohol (2.90), and isopentanol (2.88). These four compounds likely constitute the principal drivers of variation in volatile aroma profiles among the samples.

#### 3.3.8. Correlation Analysis of Physicochemical Parameters and Aroma Compounds in ‘Cabernet Sauvignon’ Wines from Different Rootstocks

Correlation analysis was performed to evaluate the relationships between the physicochemical indices and the aroma compounds in ‘Cabernet Sauvignon’ wines derived from different rootstocks. The core physicochemical parameters exhibit pronounced and highly significant interrelationships. Alcohol content, total sugar, pH, and total soluble solids are strongly and positively correlated with one another, while each is strongly and negatively correlated with total acidity. This pattern underscores the coordinated influence of grape maturity on wine composition: advanced ripening is generally accompanied by sugar accumulation and acid depletion, culminating in higher ethanol formation.

Notably, total phenolics, tannins, and total antioxidant capacity are tightly and positively correlated, indicating that phenolic constituents are the principal determinants of antioxidant activity in ‘Cabernet Sauvignon’ wine. Resveratrol content shows a significant negative correlation with volatile acidity, implying that conditions conducive to vigorous microbial activity and an elevated risk of volatile-acid accumulation may suppress stilbenoid biosynthesis (e.g., resveratrol) or compromise the stability of their glycosidic linkages.

Among the colour indices, a* (red-green axis) was highly significantly and negatively correlated with total acidity, significantly and positively correlated with pH, and highly significantly and positively correlated with anthocyanin concentration. This is consistent with the pH-dependent equilibrium of anthocyanins, the principal pigments responsible for the red-purple tonality of ‘Cabernet Sauvignon’ wines. In more acidic (lower pH) conditions, the flavylium cation—classically associated with a red hue—tends to predominate. Accordingly, lower pH would be expected to yield higher a* values and a more intensely red colour. Aldehydes were highly significantly and positively correlated with b* (yellow-blue axis), possibly reflecting the generation of aldehydic by-products during flavonoid condensation reactions occurring under oxidative or ageing conditions.

Correlations between aroma compounds and physicochemical parameters further suggest distinct regulatory controls over the formation of different volatile classes. Isoamyl acetate displayed a highly significant positive correlation with total acidity and a highly significant negative correlation with alcohol content and pH, implying that a more acidic matrix may promote acetate-ester hydrolysis or suppress volatilisation, thereby increasing measured concentrations. By contrast, ethyl hexanoate and ethyl octanoate were positively correlated with total flavonoids and dry extract, suggesting that yeast-derived fatty-acid ethyl esters may be favoured by a plentiful supply of carbon skeletons and precursors from the grape matrix. Total terpenoids were positively correlated with ethyl acetate, indicating that greater terpene abundance may coincide with reduced green-fruit notes and an enhanced pool of aromatic-ester precursors.

Within the volatile matrix itself, total ester content was strongly and positively correlated with butyl acetate and ethyl acetate, indicating that low-molecular-weight esters are the dominant contributors to overall ester levels. Ketones showed a highly significant positive correlation with isopropyl acetate, suggesting metabolic coupling between products of two pathways under conditions of amino-acid excess. Finally, the positive correlation between acetic acid and hexanal and pentanol points to shared precursors arising from fatty-acid oxidation and/or amino-acid catabolism.

#### 3.3.9. OAV Analysis of Aromatic Compounds in ‘Cabernet Sauvignon’ Wines from Different Rootstocks

**Figure 8:**
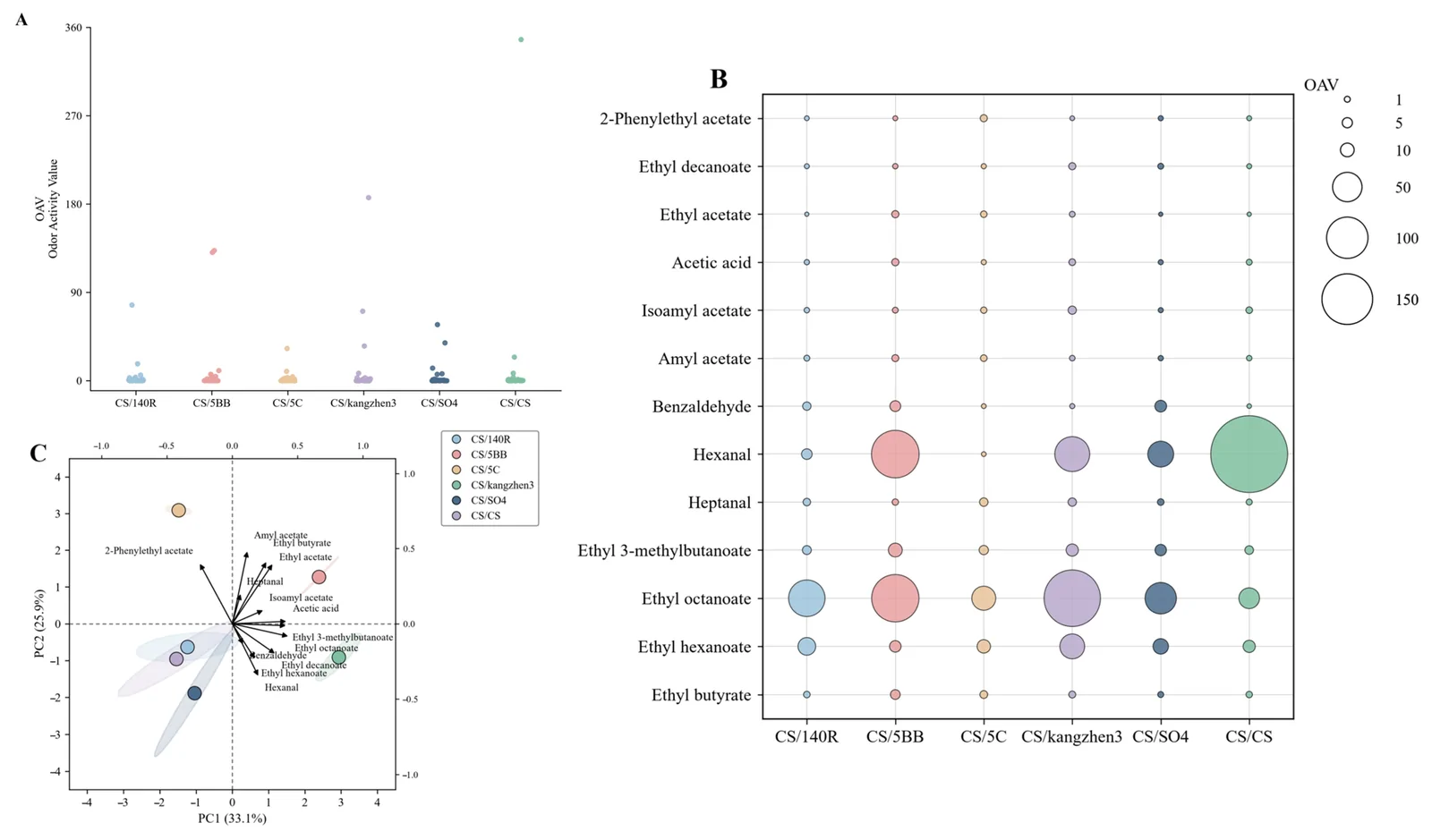
Odour activity values (OAVs) of aroma compounds in ‘Cabernet Sauvignon’ wines grafted onto different rootstocks. (**A**): OAVs of the aroma compounds; (**B**): aroma compounds with OAV > 1; (**C**): PLS biplot. Note: The shaded ellipse indicates the 95% confidence region (Hotelling’s T^2^) for the group.

To screen the potential contribution of each volatile constituent to the overall bouquet of ‘Cabernet Sauvignon’ wines produced from different rootstocks, odour activity values (OAVs) were calculated as the ratio of the semi-quantitative content of each compound to its odour threshold reported in the literature (Table 8). OAV is a useful screening indicator: an OAV > 1 indicates that a compound may contribute to the wine aroma, but it does not automatically guarantee that the compound is actually perceived as significant in a particular wine, because perception also depends on the matrix, the ethanol concentration, the assay method, and interactions with other odorants [44,45,46,47] (Figure 8A). The number of aroma compounds with OAV > 1 differed among grafting combinations: eight in CS/140R, CS/SO4, and CS/CS; nine in CS/5C and CS/kangzhen3; and ten in CS/5BB.

Across all six wines, 13 aroma compounds exhibited OAVs > 1: hexanal, heptanal, benzaldehyde, acetic acid, ethyl acetate, ethyl butyrate, isoamyl acetate, ethyl hexanoate, ethyl octanoate, ethyl decanoate, 2-phenylethyl acetate, ethyl 3-methylbutanoate, and amyl acetate. These volatiles collectively impart fruity, floral, green, and sweet notes, contributing to the distinctive aromatic profiles of the ‘Cabernet Sauvignon’ wines. Although the remaining compounds had OAVs < 1, they may still modulate the overall sensory impression, enhancing aromatic complexity and harmony. All compounds with OAV > 1 are enumerated for discussion in Figure 8B.

As depicted in Figure 8B, the compounds with the highest OAVs are predominantly esters. Esters constitute the most abundant class of volatiles in ‘Cabernet Sauvignon’ wine and are chiefly responsible for its fruity expression. In particular, ethyl butyrate, ethyl hexanoate (caproate), ethyl octanoate (caprylate), ethyl 3-methylbutanoate, and heptanal all exhibited OAVs exceeding 1 across every treatment. Ethyl butyrate conveys fruity, buttery nuances; ethyl hexanoate is associated with floral, fruity, and sweet notes; and ethyl octanoate and ethyl 3-methylbutanoate contribute floral, fruity, and sweet impressions.

Among the aldehydes, hexanal exhibited an OAV > 1 in five of the six treatments (all except CS/5C) and evokes grass, unripe fruit, and freshly cut green leaves, being unequivocally categorised as vegetal; heptanal exceeded its threshold in every treatment, whereas benzaldehyde contributed almond-like nuances in CS/140R, CS/5BB, and CS/SO4. Overall, the OAV patterns of the major volatiles were broadly comparable among wines from different rootstocks, collectively indicating a high degree of similarity in the composition of key aroma-active compounds across treatments. It should be noted, however, that OAVs are screening indicators and do not directly represent perceived aroma intensity, which is further shaped by matrix effects and odorant interactions.

To further discriminate among groups with respect to aroma compounds exhibiting OAVs > 1 and to pinpoint the principal aroma drivers, a PLS biplot was constructed (Figure 8C). In this model, CS/5BB clustered in the first quadrant, characterised by ethyl acetate, ethyl butyrate, isoamyl acetate, amyl acetate, and acetic acid. CS/5C clustered in the second quadrant, typified by 2-phenylethyl acetate. CS/140R, CS/SO4, and CS/CS grouped in the third quadrant, whereas CS/kangzhen3 was positioned in the fourth quadrant and was represented by ethyl hexanoate, ethyl octanoate, ethyl decanoate, ethyl 3-methylbutanoate, hexanal, and benzaldehyde.

## 4. Discussion

### 4.1. Effects of Rootstocks on the Physicochemical Quality and Phenolic Compounds of Wine

Fundamental physicochemical parameters are pivotal indices for assessing whether a wine conforms to quality standards, achieves sensory equilibrium, and possesses ageing potential. In this study, wines from all rootstock–scion combinations satisfied the national specifications for dry red wine, indicating that, under arid ecological conditions, each rootstock was capable of supporting normal fermentation from fully ripened fruit. Previous studies have reported that SO4 rootstocks can facilitate sugar accumulation in the scion berries, promote the build-up of secondary metabolites, and enhance the efficiency of water and mineral nutrient uptake [48,49]. In the present experiment, the CS/SO4 combination significantly outperformed the own-rooted control in dry extract, total sugar, soluble solids, and resveratrol content, and achieved the highest comprehensive principal component score. In addition, these bioactive parameters (total phenolics, total anthocyanins, resveratrol, and total antioxidant capacity) were determined at the end of the 12-month bottle-aging period, and their concentrations remained high at this sampling point, suggesting that a bottle-aging period of approximately 12 months may represent a suitable shelf-life window for retention of bioactive compounds under the present winemaking conditions. Moreover, resveratrol levels in CS/SO4 wine were significantly higher than those in the other combinations. This may be related to the generally stronger stress responses of drought-tolerant rootstocks such as SO4; however, because gene expression was not measured in the present study, this interpretation remains a hypothesis (Section 4.4). These anthocyanin- and resveratrol-rich wines are consistent with the growing consumer preference for anthocyanin-rich wines with potential health benefits [26], and they mirror recent efforts to valorise underexploited anthocyanin-rich fruits, such as karonda (*Carissa carandas* L.), through the production of alcoholic and low-alcoholic fermented beverages [27,28]. It should also be noted that all comparisons in this section are based on a single harvest year; rootstock effects of this kind can vary considerably among vintages (Section 4.4).

With respect to phenolic composition, all rootstock treatments significantly elevated total phenolics, tannins, anthocyanins, and flavonoids. Among them, CS/140R and CS/5BB exhibited higher tannin concentrations, a feature conducive to improved palate structure and enhanced ageing capacity. The accumulation of phenolic compounds is tightly linked to light exposure, temperature regime, and nitrogen availability during berry ripening [50]. Rootstocks may indirectly modulate phenolic metabolic pathways—particularly phenylpropanoid flux—by regulating nitrogen uptake and its subsequent allocation within the scion [51]. Notably, anthocyanin levels in CS/5BB and CS/SO4 were significantly higher than those in the other combinations, in agreement with their elevated a* values and greater colour saturation (c*). CIELab analysis further substantiated that CS/SO4 wines displayed a highly saturated brick-red hue, whereas CS/CS wines tended towards a less saturated rose-pink. Collectively, these results indicate that rootstocks can markedly shape wine colour by altering anthocyanin accumulation patterns in the berry skins, corroborating earlier reports on rootstock-driven colour modulation in ‘Merlot’ wines [35].

### 4.2. Regulation of Wine Aroma Compounds by Rootstocks

Aroma lies at the heart of wine’s sensory quality, and its profile and concentration are shaped by numerous factors, including berry maturity, yeast metabolism, and rootstock–scion interactions. In this study, 94 aroma compounds were identified, with the CS/5BB combination exhibiting the greatest total semi-quantitative aroma compound content. It also showed the highest ester proportion among the six rootstock–scion combinations, reaching 42.85%. Esters are pivotal contributors to the fruity and floral character of wine [52] and are produced predominantly by yeasts during fermentation via esterification between alcohols and acids [53]. Here, CS/5BB contained elevated levels of esters such as ethyl acetate, L-lactyl acetate, and ethyl butyrate, consistent with sensory scores describing this wine as having “delicate aromas and prominent fruitiness.” Consistent with earlier reports that rootstocks can modulate the form and concentration of assimilable nitrogen in the berries and thereby influence yeast metabolism and ester biosynthesis [48,54], the vigorous, stress-tolerant 5BB rootstock may have favoured ester formation in the present study. Earlier studies further reported that 5BB can significantly increase monoterpenes and C13-norisoprenoid compounds in ‘Cabernet Sauvignon’ wine [33], and the present work corroborates its particular advantage in ester enrichment.

OPLS-DA identified 15 compounds—including ethyl acetate, phenethyl alcohol, and isopentanol—as key differential aroma constituents (VIP > 1). Among them, ethyl acetate exhibited the highest VIP value (3.83), rendering it the most decisive marker for discriminating wines derived from different rootstocks. Ethyl acetate is characterised by pronounced fruity and sweet notes; its concentration peaked in the CS/5BB treatment and was comparatively lower in CS/SO4 and CS/140R. Earlier work has demonstrated that rootstocks can modulate the level of yeast-assimilable nitrogen (YAN) in berries by affecting nitrogen uptake and transport, thereby influencing the yeast’s capacity to synthesise ethyl acetate during fermentation [54]. Because yeast-assimilable nitrogen (YAN) was not directly measured in this study, these observations support a hypothesis—namely, that the 5BB rootstock may accelerate nitrogen acquisition and translocation in the scion, elevating YAN and, in turn, increasing ethyl acetate production—which should be tested directly in future work (Section 4.4). In addition, higher concentrations of phenethyl alcohol (rose-like aroma) and isopentanol (malty aroma) were detected in CS/140R and CS/5C, suggesting rootstock-dependent differences in the metabolic regulation of higher alcohols, in line with the observations of Tedesco et al. [55].

OAV analysis further pinpointed 13 compounds that contributed substantially to the overall aroma profile (OAV > 1), the majority of which were esters. Ethyl butyrate, ethyl hexanoate (caproate), ethyl octanoate (caprylate), ethyl 3-methylbutanoate, and heptanal exhibited OAVs above 1 across all treatments, imparting fruity, floral, and sweet attributes to the wines. Notably, the CS/kangzhen3 combination showed higher OAVs for isoamyl acetate (banana note), ethyl hexanoate, ethyl octanoate, ethyl decanoate, and ethyl 3-methylbutanoate, whereas CS/5BB was comparatively enriched in ethyl acetate, ethyl butyrate, and amyl acetate (fruity nuances) and CS/5C in 2-phenylethyl acetate (rose-like nuance). By contrast, CS/CS displayed the highest OAV for hexanal (grassy, green notes), consistent with the sensory descriptors of “herbal and chemical flavours” associated with this wine. The PLS biplot (Figure 8C) separated the rootstock combinations into four flavour quadrants, clearly illustrating distinct aromatic styles: CS/5BB inclined towards fruity and sweet notes, CS/5C towards floral notes, and CS/kangzhen3 towards fruity and fatty notes, while CS/140R, CS/SO4, and CS/CS expressed comparatively weaker, more vegetal characteristics. These patterns mirror those reported by Beris et al. [15] in Assyrtiko wines produced from different rootstocks. Overall, rootstock selection influences not only the total semi-quantitative content of aroma compounds but also the wine’s stylistic trajectory, offering valuable guidance for tailoring diverse wine styles. It should be stressed, however, that OAV-based conclusions are screening-level, since perceived aroma is also shaped by matrix effects and odorant interactions (Section 3.3.9).

### 4.3. Potential Physiological Mechanisms of Rootstock Regulation on Aroma Formation

The rootstock’s influence on scion aroma development is a multifaceted physiological and metabolic phenomenon, encompassing water acquisition, mineral nutrition, hormonal signalling, and the control of berry ripening. It must be emphasised that the mechanisms discussed below were not directly measured in the present study—vine water status, yeast-assimilable nitrogen, enzyme activities, gene expression, and the grape microbiome were not assessed—and they are therefore presented as literature-based hypotheses that warrant targeted verification. In this study, CS/SO4 and CS/5BB outperformed self-grafted vines in soluble solids, total sugars, and total acidity, indicating that these two rootstocks can hasten ripening while preserving a favourable sugar–acid balance, consistent with the findings of Li et al. [25]. Greater ripeness is typically accompanied by the accumulation of fatty acids, amino acids, and terpenoid precursors in the berries, thereby supplying abundant substrates for the formation of esters and higher alcohols. Conversely, rootstocks can affect the chemical form of nitrogen taken up by the roots (e.g., the ammonium-to-nitrate ratio) by shaping the structure and activity of the rhizosphere microbial community, which in turn alters the composition of assimilable nitrogen in the fruit [56]. In this work, the elevated volatile acidity observed in the CS/CS combination may be associated with the build-up of volatile acids or sulfide-derived off-notes; whether this reflects insufficient YAN remains to be verified, as YAN was not measured.

Moreover, this study showed that citronellol and nerolidol levels in CS/5BB were significantly higher than those in the other combinations; these two terpenes are among the principal contributors to the varietal aroma of ‘Cabernet Sauvignon’. Prior research has demonstrated that drought stress can induce TPS gene expression, and that rootstocks differ in the efficiency with which they transduce stress signals [57]. Accordingly, the higher terpenoid content observed in CS/5BB may be attributable to its superior drought tolerance and more effective stress-signal transduction, although this remains a hypothesis, as plant water status and gene expression were not monitored in this study.

### 4.4. Comparison of the Present Results with Previous Studies

To facilitate comparison of the present findings with the literature, the main results of this study and of previous rootstock investigations are summarised in Table 9. In general, the rootstock-dependent effects on berry and wine composition observed here are consistent with earlier reports on ‘Cabernet Sauvignon’ [32,33], ‘Merlot’ [24,35], ‘Pinot Noir’ [16,51], and ‘Assyrtiko’ [15] wines, as well as with a recent sensory-based evaluation of rootstock effects on ‘Cabernet Sauvignon’ wine aroma [34]. For instance, the rootstock-driven increases in anthocyanins and wine colour intensity observed for CS/5BB and CS/SO4 agree with the colour modulation reported by Gutiérrez-Gamboa et al. [35], whereas the rootstock-dependent shifts in the volatile profile are in line with the observations of Carrasco-Quiroz et al. [24], Li et al. [25], and Wang et al. [33]. At the same time, direct numerical comparisons between studies remain difficult because of differences in variety, vintage, site, climate, and winemaking protocols. Rootstock effects on grape and wine composition are strongly dependent on vintage and environmental conditions, as demonstrated by recent multi-year studies of ‘Cabernet Sauvignon’ [21,34]; accordingly, the present single-site, single-vintage results should not be extrapolated to other years or regions without further evidence. Several limitations of this study should also be acknowledged: (i) the experiment covered only one vineyard site and one harvest year; (ii) because the six rootstock–scion combinations reached 20 °Brix on different dates, harvest-date effects cannot be fully separated from rootstock effects; (iii) the volatile data are semi-quantitative (Section 2.2.7); (iv) OAV-based inferences are screening-level and not strictly additive (Section 3.3.9); (v) vine water status, yeast-assimilable nitrogen, enzyme activity, gene expression, and the grape microbiome were not measured, so the proposed mechanisms remain hypotheses; and (vi) sensory evaluation was performed by a small panel of four tasters in a single session. Multi-year, multi-site trials and scale-up studies are therefore needed to confirm the robustness and general applicability of the observed rootstock effects [10,19].

## 5. Conclusions

This study systematically assessed the effects of five rootstock types on the physicochemical attributes, colour, phenolic composition, and aroma profile of ‘Cabernet Sauvignon’ wine. The findings demonstrate that rootstock choice markedly modulates both the wine’s physicochemical properties and its flavour expression. CS/SO4 delivered the best overall performance and the highest principal-component score: dry extract, total sugar, soluble solids, and resveratrol were all significantly higher than in the self-grafted control, yielding a well-balanced mouthfeel and promising ageing potential. The CS/5BB combination ranked first for total semi-quantitative aroma compound content and ester abundance, showing enrichment in fruity esters such as ethyl acetate and conferring a pronounced floral–fruity bouquet. OPLS-DA coupled with OAV analysis identified 13 pivotal aroma compounds and 15 discriminatory markers, clearly differentiating the flavour signatures of each rootstock–scion pairing. Overall, SO4 and 5BB exhibit distinct advantages in enhancing body structure and aromatic complexity, respectively, providing a preliminary basis for rootstock selection and the modulation of wine style in arid regions. Because the present data were collected from a single site and a single vintage, these conclusions should be validated in multi-year and multi-site trials. In addition, scale-up studies of the most promising rootstock–scion combinations (e.g., CS/SO4 and CS/5BB) at pilot or commercial scale are warranted to validate the reproducibility of the present results under industrial conditions.

## Figures and Tables

**Table 1:** Information on rootstock varieties.

Rootstock Name	Source	Parent	Rootstock Characteristics
5BB	Austria	*V. berlandieri* × *V. riparia*	Vigorous growth, resistance to phylloxera, high rooting rate, and poor grafting compatibility.
140R	Italy	*V. riparia* × *V. rupestris*	Vigorous growth, highly drought-tolerant, phylloxera-resistant, but with inconsistent graft compatibility.
5C	Hungary	*V. berlandieri* × *V. riparia*	Moderate to strong growth, drought-resistant, phylloxera-resistant, tolerant of calcareous soils, and good graft compatibility.
SO4	Germany	*V. berlandieri* × *V. riparia*	Vigorous growth, drought resistance, strong phylloxera resistance, and wide soil adaptability.
kangzhen3	China	*V. berlandieri* × *V. riparia*	Vigorous growth, salt and alkali tolerance, phylloxera resistance, high yield, and wide soil adaptability.

**Table 2:** Morphological characteristics of ‘Cabernet Sauvignon’ grape clusters and berries at harvest.

Parameter	Description/Value
Cluster shape	Cylindro-conical, moderately compact
Cluster length	12–17 cm
Cluster Width	6–9 cm
Cluster weight	90–160 g
Berry shape	Spherical to slightly ovoid
Berry diameter	11.5–13.5 mm
Berry weight	1.1–1.5 g
Skin colour	Deep blue-black with abundant epicuticular wax bloom
Skin thickness	Medium to thick
Juice yield	Approximately 65–72% (*v*/*w*)
Seeds per berry	2–3
Pedicel length	4–6 mm
Pedicel attachment	Firm; berries resist detachment
Main harvest period	11–16 October 2020 (Shihezi, Xinjiang)

**Table 3:** Principal component analysis of ‘Cabernet Sauvignon’ grafted onto different rootstocks.

Parameter	PC1	PC2	PC3	PC4
Latent root	9.014	2.308	1.434	1.128
Contribution rate/%	60.094	15.388	9.560	7.622
Cumulative contribution rate/%	60.094	75.482	85.042	92.564
Alcohol content	0.623	0.411	0.635	0.152
Total sugar content	0.742	0.478	0.408	−0.042
Total acidity	−0.787	−0.455	−0.141	−0.315
Dry extract content	0.322	0.845	0.229	−0.335
pH	−0.893	−0.180	−0.223	−0.213
Total phenols	0.448	0.627	0.370	0.371
Total tannins	0.214	0.478	0.465	0.654
Anthocyanins	0.841	0.139	0.188	0.447
Total flavonoids	−0.060	0.944	0.122	0.164
Resveratrol	0.930	0.263	−0.137	0.102
Total soluble solids	0.740	0.246	0.091	0.397
Volatile acidity	0.915	−0.147	0.300	−0.005
Total SO_2_	0.130	0.188	0.954	0.019
Total antioxidant capacity	0.300	0.835	0.100	0.180
Vitamin C content	−0.297	0.014	0.050	−0.922

**Table 4:** Principal component scores of ‘Cabernet Sauvignon’ grafted onto different rootstocks.

Rootstock–Scion Combination	PC1 Score	PC2 Score	PC3 Score	PC4 Score	Overall Score	Ranking
CS/140R	2.554	0.536	0.308	0.054	3.453	3
CS/5BB	2.66	0.473	0.226	0.14	3.499	2
CS/5C	0.994	0.346	0.103	−0.017	1.427	5
CS/kangzhen3	0.688	0.559	0.212	0.016	1.475	4
CS/SO4	3.136	0.687	0.29	0.054	4.167	1
CS/CS	−0.932	−0.048	0.027	−0.101	−1.055	6

**Table 5:** Color differences of ‘Cabernet Sauvignon’ wine grafted onto different rootstocks.

Rootstock–Scion Combination	L*	a*	b*	c*	*h*°	Δ*E*
CS/140R	57.18 ± 0.16 b	43.33 ± 0.56 c	14.11 ± 0.24 c	45.57 ± 0.50 c	18.04 ± 0.44 c	5.85 ± 0.48 d
CS/5BB	55.55 ± 0.77 c	44.23 ± 0.46 b	16.25 ± 0.24 b	47.12 ± 0.41 b	20.18 ± 0.39 a	8.06 ± 0.22 b
CS/5C	55.21 ± 0.12 c	41.96 ± 0.25 d	12.47 ± 0.22 e	43.77 ± 0.18 d	16.55 ± 0.36 d	6.51 ± 0.25 c
CS/kangzhen3	56.01 ± 0.22 c	40.87 ± 0.45 e	11.95 ± 0.18 f	42.58 ± 0.43 e	16.29 ± 0.29 d	5.46 ± 0.30 d
CS/SO4	53.98 ± 0.54 d	47.91 ± 0.34 a	17.01 ± 0.31 a	50.84 ± 0.40 a	19.54 ± 0.26 ab	11.91 ± 0.50 a
CS/CS	60.76 ± 1.02 a	38.74 ± 0.26 f	13.56 ± 0.40 d	41.05 ± 0.15 f	19.29 ± 0.63 b	0.89 ± 0.29 e

Note: Different letters indicate significant differences among treatments (Tukey’s test, *p* < 0.05). Values are means ± SD (*n* = 3).

**Table 6:** Score evaluation of ‘Cabernet Sauvignon’ wine grafted onto different rootstocks.

Rootstock–Scion Combination	Total Score	Rank	Evaluation
CS/5BB	45.6	1	Bright ruby with purple tints. Supple on the palate, delicate aroma, persistent finish. The wine lacks overall balance.
CS/SO4	43.8	4	Clear ruby with purple hues. Soft on the palate, subtle aroma with a slight off-note, long finish. The wine is well-integrated and balanced.
CS/140R	44.8	2	Vivid ruby with purple reflections. Velvety texture, mild aroma with a faint off-odor, short finish. Unbalanced.
CS/5C	42.9	5	Glossy ruby with purple tones. Pronounced volatile acidity and chlorophenolic notes dominate.
CS/kangzhen3	44.1	3	Bright ruby with purple tints. Initially soft, but marked by volatile acidity and chlorophenolic character.
CS/CS	41.0	6	Ruby with a purplish hue. A soft entry overshadowed by volatile acidity and intense chlorophenolic notes.

**Table 7:** Semi-quantitative contents of aroma compounds in ‘Cabernet Sauvignon’ wine grafted onto different rootstocks.

Rootstock–Scion Combination	Esters (μg·L^−1^)	Esters (%)	Alcohols (μg·L^−1^)	Alcohols (%)	Acids (μg·L^−1^)	Acids (%)	Aldehydes (μg·L^−1^)	Aldehydes (%)	Ketones (μg·L^−1^)	Ketones (%)	Terpenes (μg·L^−1^)	Terpenes (%)	Total (μg·L^−1^)
CS/140R	7834.19 ± 6.57 d	14.79%	43,394.86 ± 4.60 a	81.92%	206.51 ± 1.08 d	0.39%	1201.39 ± 0.35 d	2.27%	197.72 ± 0.30 c	0.37%	138.73 ± 0.84 f	0.26%	52,973.39 ± 11.14 b
CS/5BB	22,827.76 ± 4.70 a	42.85%	26,808.05 ± 3.11 f	50.32%	468.02 ± 1.60 a	0.88%	2795.63 ± 0.46 a	5.25%	197.71 ± 0.52 c	0.37%	181.15 ± 0.82 a	0.34%	53,278.32 ± 8.17 a
CS/5C	18,729.84 ± 8.19 b	38.61%	28,229.87 ± 6.48 e	58.20%	179.96 ± 0.03 e	0.37%	344.85 ± 0.31 f	0.71%	855.11 ± 0.33 a	1.76%	167.27 ± 0.85 b	0.34%	48,506.9 ± 10.48 d
CS/kangzhen3	14,649.41 ± 4.40 c	27.98%	36,371.22 ± 2.45 c	69.47%	442.38 ± 0.45 b	0.84%	637.39 ± 0.44 e	1.22%	100.43 ± 0.06 e	0.19%	154.21 ± 0.58 d	0.29%	52,355.05 ± 6.76 c
CS/SO4	6166.43 ± 6.54 e	14.25%	33,819.49 ± 3.26 d	78.13%	136.13 ± 2.11 f	0.31%	2740.00 ± 0.19 b	6.33%	263.19 ± 0.41 b	0.61%	158.71 ± 0.74 c	0.37%	43,283.95 ± 6.85 f
CS/CS	5128.54 ± 4.40 f	10.98%	39,290.61 ± 4.40 b	84.09%	242.75 ± 1.30 c	0.52%	1777.03 ± 0.60 c	3.80%	137.28 ± 0.12 d	0.29%	147.43 ± 1.39 e	0.32%	46,723.65 ± 10.88 e

Note: Values are semi-quantitative contents (μg·L^−1^) estimated by HS-SPME-GC-MS using the internal standard method (Section 2.2.7). Different letters within the same column indicate significant differences among treatments (Tukey’s test, *p* < 0.05); values are means ± SD (*n* = 3).

**Table 8:** Odour thresholds of the aroma compounds with OAV > 1 in ‘Cabernet Sauvignon’ wines grafted onto different rootstocks.

Compound	Odor Descriptor	Odor Threshold (μg·L^−1^)	Matrix	Source
Hexanal	Grassy, green	4.5	Water	Guth [45]
Heptanal	Fatty, green	3	Water	van Gemert [47]
Benzaldehyde	Almond, sweet	350	Water	van Gemert [47]
Acetic acid	Vinegar, sour	200,000	10% ethanol (model wine)	Ferreira et al. [44]
Ethyl acetate	Fruity, sweet	7500	10% ethanol (model wine)	Ferreira et al. [44]
Ethyl butyrate	Fruity, buttery	20	10% ethanol (model wine)	Ferreira et al. [44]
Isoamyl acetate	Banana, fruity	30	10% ethanol (model wine)	Ferreira et al. [44]
Ethyl hexanoate	Floral, fruity, sweet	14	10% ethanol (model wine)	Ferreira et al. [44]
Ethyl octanoate	Floral, fruity	5	10% ethanol (model wine)	Ferreira et al. [44]
Ethyl decanoate	Fruity, fatty	200	10% ethanol (model wine)	Ferreira et al. [44]
2-Phenylethyl acetate	Rose, honey, floral	250	10% ethanol (model wine)	Ferreira et al. [44]
Ethyl 3-methylbutanoate	Fruity, sweet	3	Water	Guth [45]
Amyl acetate	Banana, pear, fruity	43	10% ethanol (model wine)	Ferreira et al. [44]

Note: Odour thresholds were compiled from the literature (values for water or hydro-alcoholic solutions commonly used in wine OAV studies) [44,45,47].

**Table 9:** Comparison of the main results of the present study with those of previous rootstock studies.

Study	Variety	Rootstocks Examined	Main Findings	Comparison with This Study
Present study	Cabernet Sauvignon	5BB, 140R, 5C, SO4, kangzhen3 vs. self-grafted	SO4 gave the best physicochemical quality (highest dry extract, total sugar, soluble solids and resveratrol); 5BB gave the highest total semi-quantitative aroma compound content and ester proportion; all wines met the national dry red wine standard	—
Wang et al. [33]	Cabernet Sauvignon	5BB, 101-14, 1103P vs. own-rooted	Rootstock altered vine growth, berry ripening, flavonoids and aromatic profiles	Consistent: rootstocks modulate phenolic and aroma composition
Zhang et al. [32]	Cabernet Sauvignon	Multiple rootstocks	Rootstock-driven accumulation of berry-skin phenolics via metabolome and transcriptome changes	Consistent: phenolic enrichment in scion berries
Farris et al. [34]	Cabernet Sauvignon	Multiple rootstocks	Rootstock shaped the aromatic expression of wine assessed by two sensory methods	Consistent: aroma style differs among rootstocks
Gutiérrez-Gamboa et al. [35]	Merlot	Multiple rootstocks	Rootstock altered grape anthocyanins, proanthocyanidins, wine colour and phenolics	Consistent: wine colour modulation through anthocyanins
Carrasco-Quiroz et al. [24]	Merlot	Multiple rootstocks	Rootstock affected the volatile composition of wines	Consistent: volatile composition modulated by rootstock
Li et al. [25]	Merlot/Marselan	Multiple rootstocks	Rootstock regulated aroma components and related gene expression	Consistent: aroma-related regulation by rootstock
Beris et al. [15]	Assyrtiko	Multiple rootstocks	Rootstock influenced nitrogen levels, must and wine composition, and sensory characteristics	Consistent: wine composition and sensory attributes affected
Chen et al. [16]	Pinot Noir	Multiple rootstocks	Rootstock affected berry and wine composition	Consistent: berry and wine composition altered
de Amorim et al. [19]	Arinto/Fernão Pires	Multiple rootstocks	Season, rootstock and cultivar influenced the volatile profiles of white wines	Consistent; highlights the dependence of rootstock effects on vintage and environment

## Data Availability

The original contributions presented in this study are included in the article and its Supplementary Materials. Further inquiries can be directed to the corresponding authors.

## Supplementary Materials

The following supporting information can be downloaded at: https://www.mdpi.com/article/10.3390/foods15183233/s1, Table S1: Linear retention indices and semi-quantitative contents of all volatile compounds identified in ‘Cabernet Sauvignon’ wines grafted onto different rootstocks; Table S2: Microbial counts at the end of the aging period.
